# Medical Residents, the Group and the Formation of Professional Identity During the COVID-19 Pandemic

**DOI:** 10.1055/s-0042-1745792

**Published:** 2022-06-29

**Authors:** Francisco Arsego de Oliveira, Helena von Eye Corleta, Edison Capp

**Affiliations:** 1 Universidade Federal do Rio Grande do Sul, Porto Alegre, RS, Brazil

**Keywords:** medical residency, professional identity, educational environment, residência médica, identidade profissional, ambiente educacional

## Abstract

Residency is still considered the gold standard for quality medical training, and acquiring a professional identity as a specialist is one of its central elements. Residents obtain this identity through both the educational environment and direct interaction with peers and supervisors. However, modifications in health care and educational routines during the recent coronavirus disease 2019 (COVID-19) pandemic have significantly impaired these channels. This study is part of a qualitative research project to analyze professional identity formation in a medical residency program in obstetrics and gynecology at a public hospital in southern Brazil. The authors conducted 28 semi-structured interviews with medical residents and preceptors, as well as a focus group with the residents, which was recorded, transcribed, and analyzed in an effort to construct major analytical categories. Restricted movement and physical contact have forced the use of alternative means of interpersonal interaction, such as communication through social media or instant messaging applications. This has also affected educational activities, such as morning rounds, lectures, and seminars. These changes represent a significant impact, especially in Brazil, where physical proximity is an important cultural feature, even in the work and school environments. We speculate that this new type of virtual interaction will also affect the formation of professional identity among obstetrician-gynecologists. These findings suggest that medical residency programs should be attentive to changes in resident training to ensure that the specialist profile and the expected skills, which are consolidated over many years, are not lost.

## Resumo

A residência médica ainda é considerada o padrão-ouro para a formação médica de qualidade, sendo o processo de construção da identidade profissional de um médico especialista um de seus elementos centrais. Os residentes obtêm essa identidade, entre outros fatores, por meio do ambiente educacional e da interação direta com colegas e supervisores. No entanto, as modificações nas rotinas assistenciais e educacionais durante a recente pandemia de *coronavirus disease 2019* (Covid-19) prejudicaram significativamente esses canais. Este estudo faz parte de um projeto de pesquisa qualitativa com o objetivo de analisar a formação da identidade profissional em um programa de residência médica em ginecologia e obstetrícia em um hospital público do sul do Brasil. Os autores realizaram 28 entrevistas semiestruturadas com médicos residentes e preceptores, bem como um grupo focal com residentes. Tanto as entrevistas como as reuniões com o grupo focal foram gravadas, transcritas e analisadas no esforço de construir categorias analíticas. Foi identificado que o movimento restrito e o contato físico forçaram o uso de meios alternativos de interação interpessoal, como a comunicação por meio de mídias sociais ou aplicativos de mensagens instantâneas. Isso também afetou as atividades educacionais, como as rounds, palestras e seminários. Essas mudanças representam um impacto significativo, principalmente no Brasil, onde a proximidade física é uma importante característica cultural, mesmo em ambientes de trabalho e de estudo. Conjectura-se que esse novo tipo de interação virtual também afetará a formação da identidade profissional entre os ginecologistas-obstetras. Esses achados sugerem que os programas de residência médica devem estar atentos às mudanças na formação dos residentes para garantir que o perfil do especialista e as competências esperadas, consolidadas ao longo de muitos anos, não sejam perdidos.

## Introduction

Due to the growing complexity of health care, physician training has been modified to include the skills necessary for new and constantly changing scenarios. 1 2 The coronavirus disease 2019 (COVID-19) pandemic represents one of the most important health crises in recent human history. In medical education, these changes have forced adaptations in curriculum design regarding new skills, interpersonal relationships, teaching strategies, and the need for community interventions. 3 4 For physicians in training, such as medical residents, the pandemic has also led to direct changes in work and leisure routines, social interaction, and their own health care. In addition to the inevitable stress that arises from training in a medical specialty, these young physicians are overwhelmed with concerns about how to address the health care of the population and how to deal with the dangers of contagion, both for themselves and those close to them. 5 6 In other words, in addition to resident burnout, which has already reached epidemic levels in recent years, the pandemic has added a further destabilizing factor. 7 8 9 10

Recently, professional identity formation has been studied as a crucial element in the long trajectory of medical education. 11 Thus, *becoming a doctor* in such a disruptive and uncertain context may deserve greater attention by researchers committed to medical education.

This study is part of a broader, qualitative research project that seeks to analyze the formation of professional identity among gynecology-obstetrics residents in a public hospital in Brazil. Medical residents and preceptors in this program were interviewed, and their interviews were recorded, transcribed, and analyzed using NVivo software (QSR International, Doncaster, Australia). This study was approved by the institutional research ethics committee (protocol no. CAAE 27172919.6.0000.5327).

After some initial interviews, it was clear that the pandemic had definitely affected this process.

## Healthcare and Educational Changes in Brazil due to the Pandemic

Following its spread from China, the COVID-19 pandemic hit Brazil in late February 2020, with the first case confirmed on February 26, and the first death recorded on March 17. Although there were enormous contrasts in the epidemiological characteristics of the disease's progress, all regions of the country were severely affected, and there were significant changes in the functioning of healthcare networks. In view of this situation, the National Medical Residency Commission (CNRM, an organ linked to the Ministry of Education that is responsible for regulating medical residency) issued a technical note on May 2020 featuring recommendations regarding the development of residence program activities during the COVID-19 pandemic. 12 In general, this document has guided the medical residency commissions of each institution and the State Medical Residency Commissions (CEREMs) about how to make residency activities more flexible to minimize the harmful effects to physicians during the specialization process. At the same time, it called on medical residents of all specialties to actively engage in health care activities aimed at the pandemic in their cities. In the same vein, the Brazilian Federation of Obstetrics and Gynecology Associations (FEBRASGO), a scientific entity that represents Brazilian gynecologists and obstetricians and is involved in the training of specialists in the area, issued its own recommendations seeking, among other points, to minimize the loss of surgical skills due to the pandemic. 13

The Hospital de Clínicas de Porto Alegre, a university hospital, is one of the largest centers for training medical specialists in southern Brazil. The institution had been preparing for the pandemic since January 2020, establishing contingency plans with staggered restrictions for assistance activities, including specific flows for each stage according to criteria that considered the number of occupied hospital beds and the spread of the disease in the state of Rio Grande do Sul and the city of Porto Alegre, where this study was conducted. The first officially registered case of severe acute respiratory syndrome coronavirus 2 (SARS-CoV-2) infection at the institution occurred on March 11, 2020, and the first death, 2 weeks later.

Since residence programs generally begin in early March, the new residents arrived at the height of all this activity. Although the official reception was still festive, including hundreds of photos shared on social media, the hospital moved to its first contingency level on March 15, limiting a number of assistance activities and the circulation of people through the hospital, which affected the patients and the medical teams. As the pandemic progressed, restrictions were implemented in residence program teaching activities and, in conformity to local health regulations, bars, restaurants, and other public places were closed to reduce crowds.

## Professional Identity: The Case of Obstetrics and Gynecology Residency

This exceptional scenario has had repercussions even on specialties that are not traditionally involved in epidemiological problems. Obstetrics and gynecology, for example, is essentially a medical specialty in women's health. The competency matrix, which serves as a guide for the almost 300 residency programs in obstetrics and gynecology in Brazil, was defined through recent CNRM legislation. 14 This detailed document divides the competences to be achieved into several axes of care according to each year of the residency. Although it does not establish a minimum number of expected procedures, there is a clear indication that the resident must master certain techniques, which presupposes having acquired such competence through exhaustive training.

As in any other surgical specialty, it requires, in addition to knowledge and attitudes, the development of a series of motor skills, which residents obtain through continuous exposure to a significant and varied number of situations in which they can exercise their clinical reasoning in an appropriate, independent, and safe way for their patient and themselves. 15

In this complex trajectory towards specialized medical practice, the interrelationship of peers plays a crucial role. Although the formation of a physician's professional identity involves an eminently individual path, it always occurs alongside others who are undergoing the same situation. For newcomers, being accepted into a peer community that has a structured professional identity can pose additional stress during the transition from student to doctor. This is true among the residents and the health team members they relate to professionally. In other words, becoming a doctor is also directly linked to the process of appearing like a doctor to other people. Thus, forming a group of residents has been a hallmark of medical residency since its beginning. 16 In other words, the formation of professional identity is a two-way street: from the inside out (the doctor must *feel* like a doctor) and from the outside in (other people must *see* him or her as a doctor).

Although the millennials' way of learning has led to changes in teaching and medical residency in recent years, some traits, such as group support, have remained equally important despite generational differences and increasing technological sophistication.

Regardless of preparation, which can begin prior to entering medical school, the transition from being a student to a resident is still a huge challenge in any specialty. 17 Despite previous familiarity with some of the activities of medical school, being registered with state agencies and “having the seal”, that is, formal authorization to practice medicine, puts professional activity in a different perspective.

Likewise, coexistence and relationships with new colleagues—some completely unknown and others who were former competitors for a place in the program—is an important element in this equation. Interaction between colleagues in outpatient clinics, in the operating room, on duty and in seminars is not only inevitable, but it can also help residents bear the moments of stress and emotional overload that occur during the program. Often, it is this type of connection that allows young doctors to satisfactorily reach the end of their journey toward obtaining the title of specialist after finishing the residency program.

Following the rapid expansion of information, communication, and technology resources, there seems to be no doubt that this generation has a different learning style than its predecessors. 18 19 20 Nevertheless, despite such autonomous learning and skill development, interpersonal relationships and collaborative work among peers remain among the most important pillars in resident training, although different programs do not always recognize them in the same way. 21

## The Particularities of Brazilian Programs

The medical field in Brazil reflects certain particularities observed in the country as a whole. Deciphering Brazilian culture has, in itself, been a constant challenge for generations of anthropologists. However, despite great regional diversity, we can say that Brazilian culture has a more permissive tendency than many other countries, both in romantic relationships and in friendships. 22 Among other characteristics, it is a culture marked by physical proximity; for example, greetings with hugs and kisses are not unusual.

This greater permissiveness in interpersonal relations is also reflected in the work environment. In medical residencies, for example, new friends are made quickly through numerous social activities during the program, although mainly outside the formal structure. The welcoming rituals for new residents, reception parties, and other informal gatherings that occur in the first weeks of the program are good examples of this. Such events, especially among residents of the same year, become habitual and strengthen friendships and the feeling of belonging to the larger group. These feelings continue even after the residents go their separate ways as trained professionals. The testimonies of two final-year residents illustrate this dimension well:

*“Towards the end of the first year, we start working together, with two first-year residents and one second-year resident. But there was always this thing about ‘after our shift, let's go out and get something to eat, after our shift let's go out and...’”*

*“To deal with stress, I talk to my fellow residents. We have a very good group, my colleagues from the same year. We're pretty united. We try to help each other. Just yesterday, two colleagues were going through some situations and we [said] ‘Let's go have some coffee together, let's go have lunch together’, we help each other a lot. And this is in addition to family support”*

(these and the following quotes have been translated from Portuguese).

During a medical residency program, any activity can have an educational role, even informal ones and those involving interpersonal and group relationships. It is also in these environments that information about jobs, courses, publications, and articles of interest are exchanged. As Bonet 23 notes in his study on the training of family physicians in Argentina and Brazil, it is interesting to observe the different environments in which professional socialization takes place. It is in these spaces that, more or less explicitly, the transmission of values and performance standards occurs. Thus, the group of residents becomes the space in which they share their personal stories and reveal their personalities in a more open way, without the risk of being judged by preceptors. In fact, it is when residents share their problems with the group, such as difficulties with patients or conflicts within the staff, that they receive collective support. As a second-year resident reports:

*“I was having a very stressful month that was turned around by my great team. I had an episode that I guess should be called burnout, in which I found myself yelling at a patient. I understood I was in a bad environment. But my colleagues took me aside and said: ‘What's going on? Let's sit down and talk.’ And then I was able to get myself together, and the month ended very well.”*

Obviously, the group dynamics depend essentially on how these relationships are established and maintained over time. On one hand, there can be a spirit of camaraderie and cooperation, but on the other, overexposure can be dangerous because it reveals weaknesses, faults, or other behaviors that are considered out of step with the group's principles. These discrepancies, if striking, can even compromise a resident's participation in the group. This is because, despite theoretically enjoying independence, more flexible limits, and their own set of values, groups of residents assume a position of relative submission to the larger structure of the residency program. In other words, there is a permanent tension between the central core of the program and the groups of residents who, being the weak end, are “shaped” by the more consolidated and, therefore, more powerful group.

Two residents' reflections about how a colleague abandoned the program the previous year are very enlightening about this type of thinking:

*“The eight of us who entered [the program] are progressing, for better or worse, by leaps and bounds, but, when necessary, we get together and hammer things out. I think the group we have now is very good, we help each other. Still, there are, of course, differences, little intrigues, but I think that, in general, it is a supportive group. There was a resident who requested a transfer to another state, and, recently, one of our oldest residents gave up, taking a test for another specialty. And there was another one who gave up at the beginning of the year and is doing another clinical specialty. These people did not feel at home in our program.”*

*“Two colleagues ended up quitting the program. I think it was because of a number of things. I think the first one gave up because she didn't really fit in; she gave up in July. I think it's because she didn't really like what she was doing. But the second one, I think, was because of built-up pressure. She had a sick relative and the pressure her colleagues–her teammates–put on her was also great.”*

Even in this environment, interesting changes have occurred more recently regarding group formation. The growing and widespread use of technology has proven inexorable in all dimensions of our lives. Interpersonal and intergroup communication should be highlighted, especially during the pandemic, which has required physical distancing as a way to reduce disease transmission. Communication through platforms such as Facebook, Instagram, and applications—such as WhatsApp and Telegram—have become very popular, especially among young people. With these tools, unlimited instant text and voice messages, images, and videos can be exchanged virtually for free.

Thus, other forms of interpersonal communication had to be quickly created to ensure a sense of interactivity and belonging, which is essential for medical residency. *“They [only] had 2 weeks of residency! Many people can't make it to meetings. For example, what brought my group together was this: scheduling a happy hour to talk trash about the professors together, call each outer out, to get together, we did that a whole lot. So, now, we can very easily resolve our problems, trade shifts, and admonish each other. [The new residents] often have to go before the full group to ask to trade shifts.”*

In this context, there was a rush toward online communication tools and virtual meetings. Although most of these applications have been used routinely for several years, the lockdown has intensified their use. What was formerly resolved in person began happening online—even the most common questions. As one resident said: “ *WhatsApp saved us!* ” Nevertheless, she pointed out that “ *it's not the same thing, but you can still feel supported.* ”

The changes in these relationships seem to indicate adjustments in both form and content. They were creative adaptations implemented very quickly during an uncertain situation and an urgent need for interaction. Thus, a quick solution for a communication problem has now assumed the characteristics of professional identity formation.

Assessing the real impact of these changes will require, in addition to a longer follow-up time, a more comprehensive and careful perspective.
